# Androgen receptor-mediated downregulation of microRNA-221 and -222 in castration-resistant prostate cancer

**DOI:** 10.1371/journal.pone.0184166

**Published:** 2017-09-08

**Authors:** Bin Gui, Chen-Lin Hsieh, Philip W. Kantoff, Adam S. Kibel, Li Jia

**Affiliations:** 1 Division of Urology, Department of Surgery, Brigham and Women’s Hospital, Harvard Medical School, Boston, Massachusetts, United States of America; 2 Department of Medical Oncology, Dana-Farber Cancer Institute, Harvard Medical School, Boston, Massachusetts, United States of America; 3 Department of Medicine, Memorial Sloan Kettering Cancer Center, New York, New York, United States of America; Florida International University, UNITED STATES

## Abstract

MicroRNAs (miRNAs) play important roles in cancer formation and progression by suppressing the production of key functional proteins at the post-transcriptional level in a sequence-specific manner. While differential expression of miRNAs is widely observed in cancers including prostate cancer (PCa), how these miRNAs are transcriptionally regulated is largely unknown. MiRNA-221 and miRNA-222 (miR-221/-222) are well-established oncogenes and overexpressed in breast, liver, pancreas, and lung cancer, but their expression and biological functions in PCa remain controversial. Both up and down regulation have been observed in patient samples. Specifically, studies have demonstrated miR-221/-222 function as oncogenes, and promote PCa cell proliferation and the development of castration-resistant prostate cancer (CRPC). However, the expression level of miR-221/-222 is downregulated in several miRNA expression profiling studies. In this study, we demonstrate miR-221/-222 are androgen receptor (AR)-repressed genes and reside in a long primary transcript (pri-miRNA). Derepression of miR-221/-222 after androgen deprivation therapy (ADT) may enhance PCa cell proliferation potential through promoting G1/S phase transition. This function is likely transient but important in the development of CRPC. Downregulation of miR-221/-222 subsequently occurs once AR activity is restored through AR overexpression in CRPC. Our findings shed light on the complexity of transcriptional regulation of miRNAs in PCa and suggest context-dependent targeting of oncogenic miRNAs.

## Introduction

MicroRNAs (miRNAs) are small non-coding RNAs about 22 nucleotides in length that regulate the expression of target mRNA post-transcriptionally and influence a multitude of cellular processes during development and disease. Dysregulation of miRNAs has been widely observed in numerous cancers and different stages of cancer. These miRNAs function as oncogenes or tumor suppressors based on their inhibition of tumor-suppressive and oncogenic target mRNAs, respectively. Prostate cancer (PCa) is the most frequently diagnosed cancer in American men. While miRNA profiling shows that many miRNAs are differentially expressed in PCa tissues versus the corresponding normal tissues, only a small number of them have been experimentally determined to be involved in the development and progression of PCa.

MiRNA-221 and miRNA-222 (miR-221/-222) are two highly homologous miRNAs, which are clustered on the short arm of chromosome X. They are overexpressed in the majority of epithelial cancers, including breast, liver, pancreas, and lung cancer [1–4]. It is believed that miR-221/-222 play an oncogenic role in these cancers. They control cell cycle progression through inhibition of CDKN1B/p27 and CDKN1C/p57, and facilitation of G1/S phase transition [5–9]. The role of miR-221/-222 in PCa remains controversial. PCa is initially dependent on androgen for growth and sensitive to androgen deprivation therapy (ADT). However, almost all patients progress to castration-resistant prostate cancer (CRPC). CRPC remains an incurable disease through multiple resistance mechanisms to ADT [10]. Interestingly, both up and down regulation of miR-221/-222 in PCa, especially in CRPC have been reported [9, 11–21]. It is unclear how miR-221/-222 are transcriptionally regulated during PCa progression from androgen dependence to castration resistance and whether miR-221/-222 act as oncogenes or tumor suppressor genes. The variability between these studies may be related to different methodology, molecular heterogeneity and sample collection (such as tumor purity and stroma contamination). In addition, it should be noted that miRNA function is cell type-specific and context-dependent. It is possible that miR-221/-222 may act as tumor suppressor genes in a certain setting [22]. On the other hand, consistent upregulation of miR-221/-222 is observed in all epithelial cancers except PCa raises the question of whether there is a unique tissue-specific underlying molecular mechanism.

In general, a miRNA is processed from a primary transcript (referred to as pri-miRNA) that is transcribed by RNA polymerase II (Pol II). The pri-miRNA can extend hundreds of kilobases in length and include more than one precursor miRNA hairpin (pre-miRNA). Studies have shown that approximately 50% of miRNAs are intragenic and mostly located within introns of protein-coding genes [23, 24]. While about one third of intronic miRNAs are transcribed from their own promoters, the majority are co-expressed and co-regulated with the host gene in which they reside. In other words, intronic miRNAs and host mRNAs may be processed from the same primary transcript. On the other hand, intergenic pri-miRNAs are poorly characterized. There is no well-annotated intergenic pri-miRNA database. Nevertheless, similar to transcriptional regulation of protein-coding genes, intergenic pri-miRNA expression is largely controlled by transcription factors through promoters and enhancers. Promoter and enhancer regions of intergenic pri-miRNAs and protein-coding genes share many common epigenetic features, including histone modification marks. MiR-221/-222 are clustered genes separated by 726 bases based on the reference genome hg19. Since miR-221/-222 are produced from the same pri-miRNA, expression alteration of these two miRNAs was observed in cancer cells in a synchronous fashion.

Here, we present integrated genomic data at the miR-221/-222 locus. We define and characterize a pri-miRNA for miR-221/-222 in CRPC cells. We investigate whether and how miR-221/-222 are regulated by AR, which may explain the disparity of miR-221/-222 expression level in different PCa tumors. Because miR-221/-222 target key cell cycle genes and increase cellular proliferation potential in cancer cells, a complete understanding of their regulation during PCa progression may have clinical applications in the future.

## Materials and methods

### Cell culture and materials

Human prostate cancer LNCaP and C4-2B cells were described previously [25]. All cell lines were maintained in RPMI 1640 supplemented with 10% (v/v) fetal bovine serum (FBS). Antibodies are: anti-AR (Abcam, ab74272), anti-FOXA1 (Abcam, ab23738), anti-acetylated H3K9/14 (Millipore, #06–599), anti-acetylated H3K27 (Abcam, ab4729), ant-dimethyl-H3K4 (Millipore, #07–030), ant-trimethyl-H3K4 (Millipore, #07–473), anti-trimethyl-H3K27 (Millipore, #07–449), anti-Pol II (Santa Cruz Technology, sc-899), and anti-β-tubulin (Santa Cruz Technology, sc-80011).

#### Western blot

Anti-AR (1:200 dilution), anti-FOXA1 (1:1000 dilution), and anti—β-tubulin (1:2000 dilution) were used as primary antibodies in Western blot. Experiments were performed as previously described [26].

#### Cell proliferation and cell cycle assays

C4-2B cells were co-transfected with miR-221 and miR-222 precursors or inhibitors (Ambion) at a final concentration of 20 nM each using Lipofectamine RNAiMAX Transfection Reagent (Life Technologies) according to the manufacturer’s instruction. Cell number was counted 3 days after transfection. Cell cycle analysis was performed in parallel using Propidium Iodide Flow Cytometry Kit (Abcam). Cell cycle distribution of 10,000 gated cells is presented.

#### MiRNA microarray and quantitative reverse transcription polymerase chain reaction (RT-qPCR)

LNCaP and C4-2B cells were grown in RPMI 1640 supplemented with 10% FBS. Total RNA was extracted using TRIzol Reagent (Thermo Fisher Scientific) and submitted to Exiqon for miRNA expression profiling using miRNA microarrays. Dual-color experiments using Hy3^™^ and Hy5^™^ as labeling dyes with dye swap were conducted for comparison of differential expression between LNCaP and C4-2B cells. In addition, miR-221/-222 were quantified by RT-qPCR using TaqMan MicroRNA Reverse Transcription Kit (Applied Biosystems) and FastStart Universal Probe Master (Roche) according to the manufacturer’s instruction. U6 snRNA was used for normalization. All primer sets for TaqMan MicroRNA Assays were purchased from Applied Biosystems. Values are means ± standard deviations (SD) of triplicate wells.

#### RT-qPCR for mRNA expression

After treatments as indicated, total RNA from cells or tissues was extracted using TRIzol Reagent. Complementary DNA (cDNA) was prepared using the iScript cDNA Synthesis Kit (Bio-Rad), and qPCR was conducted using SYBR Green PCR Master Mix (Applied Biosystems). Triplicate PCR reactions were conducted. The primers are: Site A-forward, 5’-GTCATAATGGCAGAGTCCTCAT-3’; reverse, 5’-TACATGGCAGAAGAGCAGAAG-3’; Site B-forward, 5’-CAGAAGTTCATGGATGGGAGAG-3’; reverse, 5’-TGCTTTGTACTCTTCGGGATTAG-3’. Primers for AR, PSA, and GAPDH mRNA were previously described [27, 28].

#### Small interfering RNA (siRNA) knockdown

For AR and FOXA1 knockdown, cells were seeded in 6-well plate and transfected with gene-specific siRNA at a final concentration of 20 nM using Lipofectamine RNAiMAX Transfection Reagent according to the manufacturer’s instruction. SMARTpool siRNAs against AR, FOXA1, and non-specific (NS) control were purchased from Thermo Fisher Scientific. Cells were collected for miR-221/-222 RT-qPCR analysis 3 days after transfection.

#### Luciferase assay

Cells were transfected with miRNA luciferase reporters, pMiR-221-Luc or pMiR-222-Luc (Signosis). These two vectors are firefly luciferase-based reporter constructs, which have a unique miRNA target site at 3’UTR perfectly complementary to miR-221 and miR-222 respectively. pRL-TK Renilla luciferase reporter (Promega) was co-transfected as an internal control. Luciferase activity was measured 24 hours after transfection using Dual-Luciferase Reporter Assay System (Promega). The results are represented as Firefly/Renilla ratio.

#### Chromatin immunoprecipitation sequencing (ChIP-seq) and RNA sequencing (RNA-seq)

ChIP-seq data at the miR-221/-222 locus are from our previously generated datasets [27]. These data have been deposited in the Gene Expression Omnibus (GEO) database under accession number GSE40050. RNA-seq was performed in C4-2B cells that were grown in 5% charcoal-stripped fetal bovine serum (CSS) for 3 days. Briefly, 10 ug of total RNA was extracted and depleted of ribosomal RNA (rRNA) using the RiboMinus kit (Thermo Fisher Scientific). Library preparation and data analysis were performed as previously described [27]. RNA-seq data are available from http://epigenomics.wustl.edu/liData/C42B_riboM_1.bw.

#### Chromatin immunoprecipitation (ChIP) and formaldehyde-assisted isolation of regulatory elements (FAIRE)

ChIP experiments were performed as described previously [27]. FAIRE experiments were performed using a published protocol [29], Briefly, cells were fixed with 1% formaldehyde for 10 minutes at room temperature followed by sonication. Free DNAs at nucleosome depleted regions were purified through phenol/chloroform extraction. The DNA enrichment was analyzed by RT-qPCR. The AR binding sites and transcription start sites (TSS) were examined. The primers for the miR-221/-222 locus are: MiR-221/-222 AR-forward, 5’-TCTTTGCAATCTGAACACAGCA-3’; reverse, 5’-TGCCCGACTTCTAAGCATTAGC-3’; miR-221/-222 TSS-forward, 5’-CTCCATTAAACCCTTGTCCAAAC-3’; reverse, 5’-GGAATGGGTTTGCTGAACTTAC-3’. Primers for the PSA enhancer and promoter were described previously [28].

#### Xenograft tissue analysis

LuCaP 35 and LuCaP 35CR xenografts were established from metastatic human prostate tumors in lymph nodes [30]. LuCaP 35 (grown in intact mice), LuCaP 35C (10 days after castration), and LuCaP 35CR (grown in castrated mice) tissues were collected directly from xenograft models. LuCaP35CR was developed 4 months after castration and then maintained in castrated mice. Fresh frozen tissues were provided by Dr. Robert Vessella at University of Washington, Seattle. Total RNA was extracted using TRIzol Reagent. Gene expression was examined using RT-qPCR.

#### Microarray data analysis

MiR-221/-222 and AR expression data from 111 PCa patient samples were obtained from microRNA microarray (GSE21036) and whole exon microarray (GSE21034) datasets respectively. Patients were ranked according to AR expression level and grouped into high-AR (the first quartile, n = 28) and low-AR (the last quartile, n = 28). Differential expression of miR-221/-222 between two groups was analyzed by non-parametric Mann-Whitney test. Boxplot shows the mean ± 95% confidence interval (CI).

## Results

### MiR-221 and miR-222 are overexpressed in CRPC cells and required for CRPC cell proliferation

**Fig 1:**
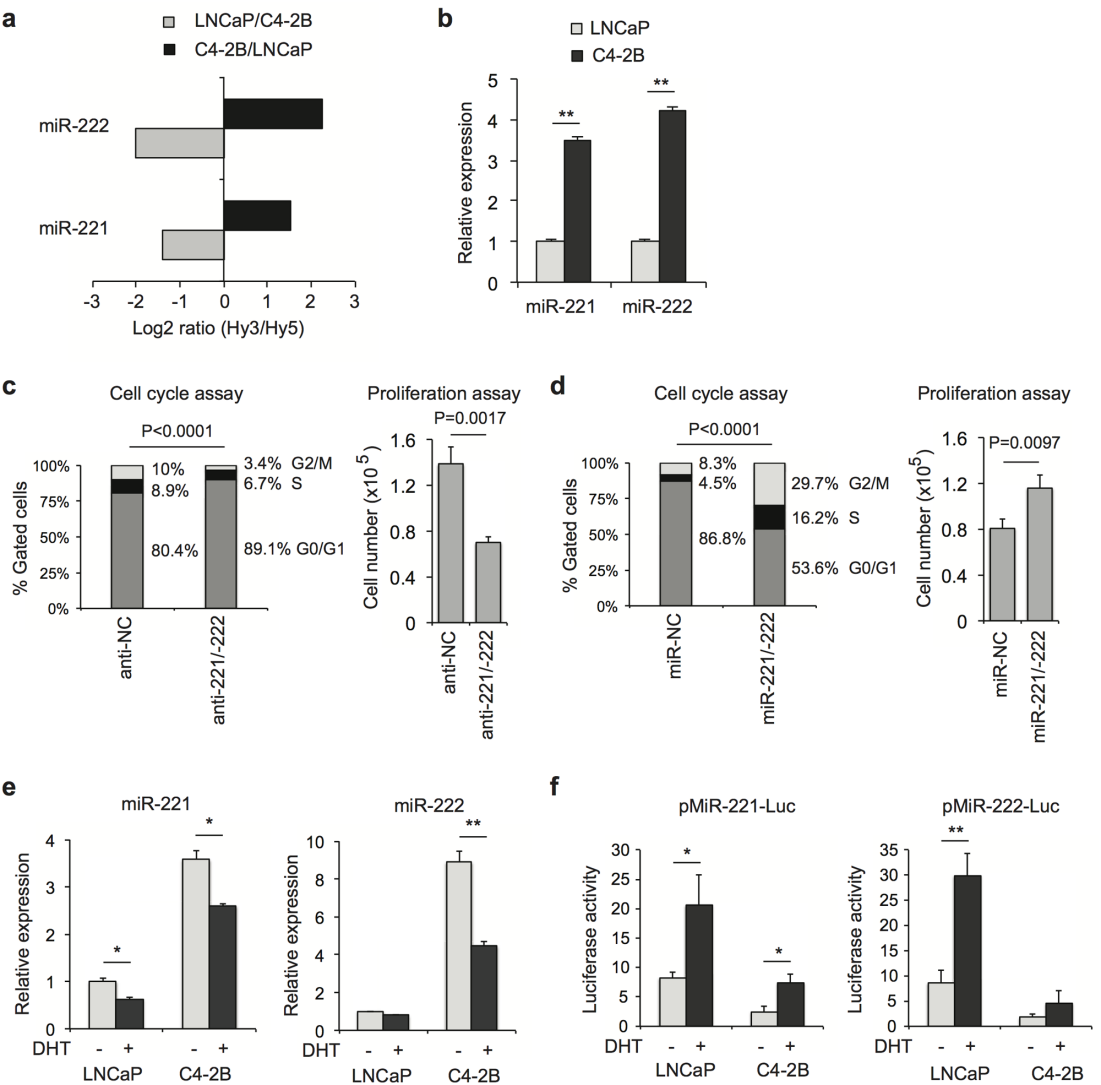
Overexpression of miR-221/-222 promotes CRPC cell proliferation. (**a**) MiRNA microarray results showing upregulation of miR-221/-222 in C4-2B cells compared to LNCaP cells. (**b**) RT-qPCR confirmed microarray results. The expression level was normalized by U6 snRNA. (**c**) C4-2B cells were co-transfected with miR-221 and miR-222 inhibitors (anti-221/-222) and non-specific control (NC). Cell number was counted 3 days after transfection. Cell cycle analysis was performed in parallel. **(d)** C4-2B cells were co-transfected with miR-221 and miR-222 precursors (miR-221/-222) and analyzed as described in (c). (**e**). LNCaP and C4-2B cells were grown in phenol red-free RPMI 1640 media containing 5% charcoal-stripped fetal bovine serum (CSS) for 3 days followed by treatment with 10 nM dihydrotestosterone (DHT) or ethanol control for 16 hours. MiR-221/-222 were examined using RT-qPCR. (f) LNCaP and C4-2B cells were grown in phenol red-free RPMI 1640 media containing 5% CSS with or without 10 nM DHT for 2 days. pMiR-221-Luc or pMiR-222-Luc luciferase constructs containing DNA sequences at 3’UTR complementary to miR-221/-222 were transfected into cells. pRL-TK Renilla luciferase reporter was co-transfected as an internal control. Luciferase activity (Firefly/Renilla ratio) was determined 24 hours after transfection. The p-value for cell cycle distribution of 10,000 gated cells was determined using a chi-squared test. The p-value for other assays was determined using a two-tailed Student’s t-test. Data presented are mean ± SD of three measurements. * P < 0.05; ** P < 0.01.

First, we conducted miRNA expression profiling in androgen-dependent LNCaP and LNCaP-derived C4-2B cells using miRNA microarray analysis (S1 Table). C4-2B is a CRPC cell line, which was generated from a bone metastasis after castration in xenograft model [31]. In line with previous studies in CRPC cells [18, 32, 33], we found that miR-221/-222 were overexpressed in C4-2B cells in contrast to LNCaP cells by approximately 3- and 4-fold respectively (Fig 1a). MiR-221/-222 are the top two miRNAs upregulated more than 2-fold, indicating their important functions in C4-2B cells. This result was further validated by RT-qPCR (Fig 1b). To test whether miR-221/-222 are required for C4-2B cell proliferation, we knocked down the expression of miR-221/-222 by transfecting commercially synthesized miR221/-222 inhibitors. Cell cycle analysis showed a significant increase of C4-2B cells in the G0/G1 phase and a corresponding decrease in the S and G2/M phases (Fig 1c). Accordingly, proliferation of C4-2B cells was significantly decreased after miR-221/-222 inhibition. In contrast, overexpressing miR-221/-222 precursors dramatically decreased cells in the G0/G1 phase and increased cells in the S phase and G2/M phases (Fig 1d). Proliferation of C4-2B cells was significantly enhanced. These results are consistent with the notion that miR-221/-222 are required for CRPC cell proliferation.

While it has been reported that miR-221/-222 are regulated by NF κB in PCa [34], androgen may also affect the production of the miR-221/-222 [35]. In this study, we found that the expression level of miR-221/-222 was significantly suppressed after androgen treatment in both LNCaP and C4-2B cells, although androgen-induced inhibition was greater in C4-2B cells (Fig 1e). To further examine the functionality of miR-221/-222 in LNCaP and C4-2B cells, the luciferase reporters carrying a complementary sequence to miR-221 or miR-222 at 3’ UTR of luciferase gene were transfected into LNCaP and C4-2B cells. As expected, we observed significantly lower luciferase activities in C4-2B cells compared to LNCaP cells (Fig 1f). Androgen treatment enhanced luciferase activities in both cells corresponding to downregulation of miR-221/-222, indicating androgen-mediated regulation.

### MiR-221 and miR-222 are AR-repressed genes

**Fig 2:**
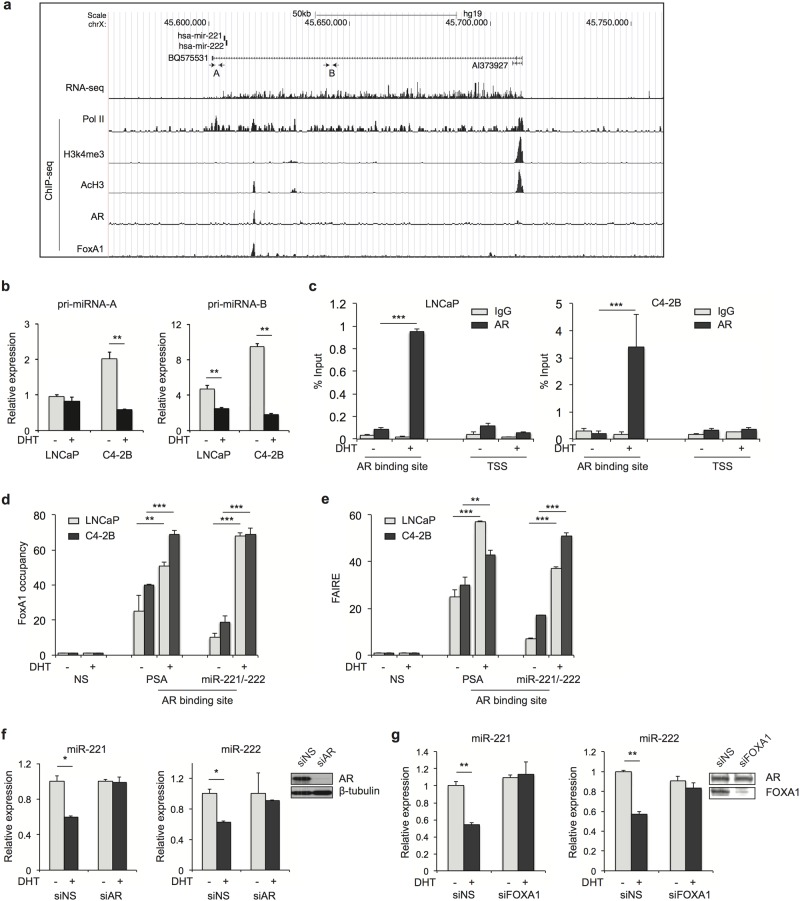
Mir-221/-222 are AR-repressed genes. (a) RNA-seq and ChIP-seq results showing genomic region around the miR-221/-222 locus. Expression of the primary transcript matches enriched Pol II signals. Robust H3K4me3 and AcH3 peaks are present at the TSS. AR binding site is observed upstream of miR-221/-222 and coincides with FOXA1 and AcH3 peaks. (**b**) The expression level of pri-miRNA was measured by RT-qPCR using two sets of primers at sites A and B. (**c**) LNCaP and C4-2B cells were grown in phenol red-free RPMI 1640 media containing 5% CSS for 3 days followed by treatment with 10 nM DHT or ethanol control for 4 hours. AR ChIP-qPCR analyses were conducted in both LNCaP and C4-2B cells. Strong DHT-induced AR occupancy was detected. (**d**) FOXA1 occupancy at AR binding sites was confirmed using ChIP-qPCR. (**e**) FAIRE assay was used to measure open chromatin structure at AR binding sites of the PSA and miR-221/-222 loci. (f) C4-2B cells were grown in RPMI 1640 medium with 5% CSS for 2 days followed by AR siRNA transfection. RT-qPCR analyses of miR-221/-222 levels were conducted 3 days after siRNA transfection. Cells were treated with 10 nM DHT or ethanol for 16 hours before RNA extraction. AR knockdown was confirmed by Western blot. (**g**) Expression of miR-221/-222 was also examined after FOXA1 knockdown. FOXA1 knockdown was confirmed by Western blot. The p-value was determined using a two-tailed Student’s t-test. Data presented are mean ± SD of three measurements. * *P* < 0.05; ** *P* < 0.01; *** P < 0.0001.

Next, we sought to determine whether and how androgen inhibits expression of miR-221/-222 through the AR at this locus. We studied this in C4-2B cells because a greater inhibitory effect was observed. It is known that miR-221/-222 are transcribed in a single primary transcript [36]. Computational analysis has predicted the length of the primary transcript of miR-221/-222 is over 10 kilobases [37]. However, the exact pri-miRNA length and TSS have not been experimentally defined. We performed RNA-seq in C4-2B cells and identified a highly expressed transcript in which miR-221/-222 reside (Fig 2a). The transcript does not code a known protein. The whole transcript was occupied by Pol II based on our ChIP-seq data generated previously [27]. We then examined the expression of this transcript by RT-qPCR using two sets of primers at sites A and B. We observed higher expression level in C4-2B cells compared to LNCaP cells, which was inhibited by androgen treatment, but more so in C4-2B cells (Fig 2b). Next, we examined histone modification marks for the promoter and found a robust peak for histone H3 acetylation (AcH3) and H3K4 tri-methylation (H3K4me3) at the TSS of the transcript (Fig 2a). Importantly, our AR ChIP-seq data showed a strong AR binding site 9.6 Kb upstream of miR-221/-222. AR occupancy was validated in both LNCaP and C4-2B cells by an independent site-specific ChIP-qPCR (Fig 2c). These analyses revealed an AR-regulated pri-miRNA in which miR-221/-222 reside.

The mechanism underlying AR-mediated transcriptional activation has been well studied, whereas AR-mediated transcriptional repression is poorly understood. FOXA1 functions as a pioneer factor, which facilitates AR recruitment to AR binding sites. Like the PSA enhancer, we detected strong pre-existing FOXA1 occupancy, which coincides with the AR binding site at the miR-221/-222 locus in the absence of androgen (Fig 2d). FOXA1 binding was further enhanced after androgen stimulation. Importantly, the AR binding site at the miR-221/-222 locus display an open chromatin structure determined by FAIRE assay (Fig 2e). The FAIRE signal intensity was significantly increased after androgen stimulation, suggesting AR-mediated repression undergoes chromatin modifications with more open structure. This is similar to the change observed at the AR enhancer region of the PSA gene. Knockdown of AR or FOXA1 diminished the AR-mediated inhibitory effect on miR-221/-222 expression in C4-2B cells (Fig 2f & 2g). Taken together, our results suggested that FOXA1 and open chromatin structure are required for AR-mediated repression.

### Specific histone modifications are associated with AR-mediated repression at the miR-221/-222 locus

**Fig 3:**
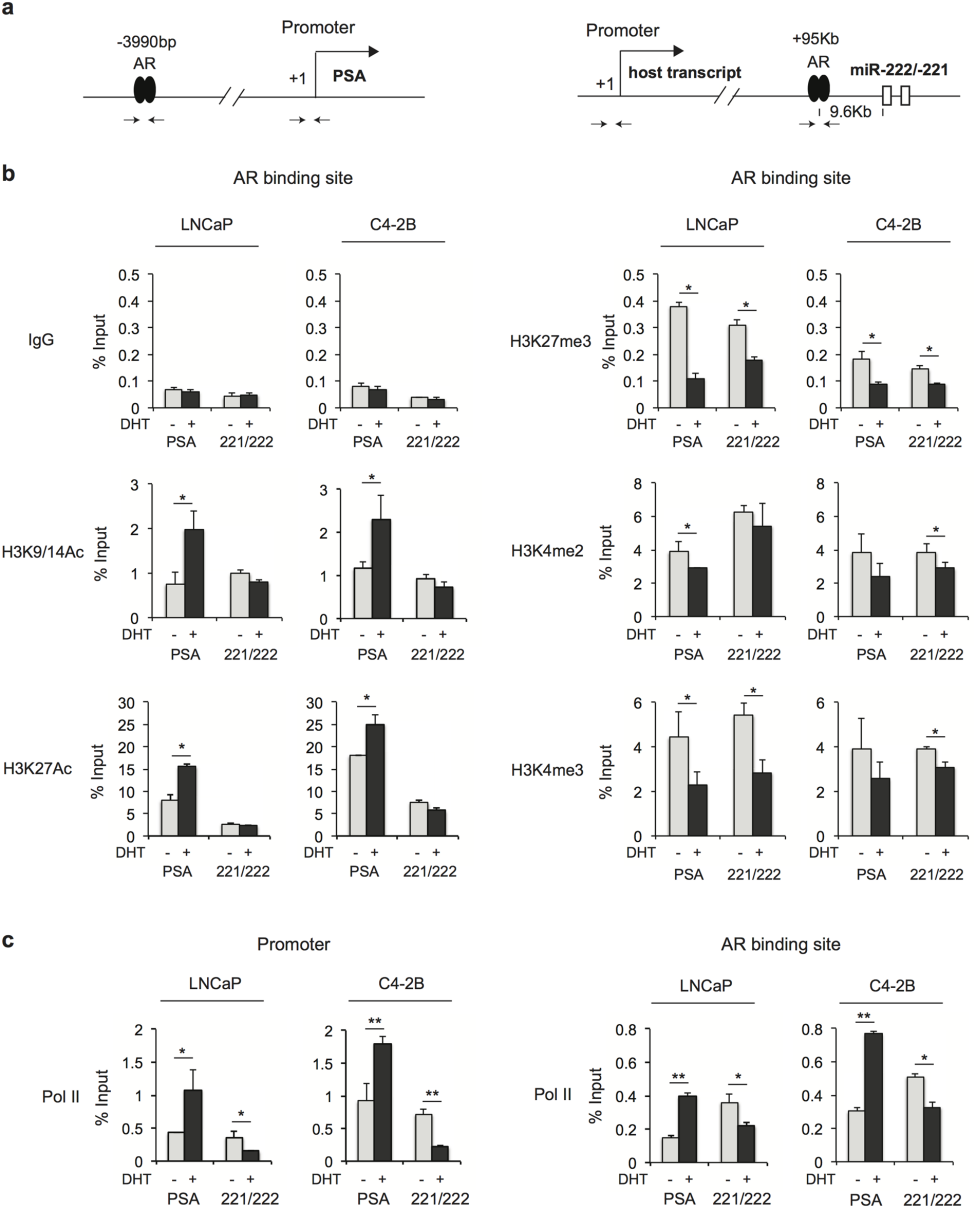
Analysis of histone modifications and Pol II enrichment at the miR-221/-222 locus versus PSA locus. (**a**) The schematic diagrams represent AR binding sites at the PSA (left) and miR-221/-222 (right) loci. (**b**) LNCaP and C4-2B cells were grown in RPMI 1640 medium with 5% CSS for 3 days followed by the treatment with 10 nM DHT or ethanol control for 4 hours. Histone H3K4 acetylation (H3K9/14Ac and H3K27Ac), H3K27 tri-methylation (H3K27me3), H3K4 di- and tri-methylations (H3K4me2 and H3K4me3) were examined by ChIP-qPCR at AR binding sites as indicated. (**c**) LNCaP and C4-2B cells were treated as described in (b). Pol II enrichment at promoters and AR binding sites were examined by ChIP-qPCR. The p-value was determined using a two-tailed Student’s t-test. Data presented are mean ± SD of three measurements. * *P* < 0.05; ** *P* < 0.01.

AR binding sites can be epigenetically marked by specific histone modifications and fluctuation of these modifications has been linked to transcriptional regulation. To understand chromatin modifications associated with AR-mediated activation versus repression, we examined several specific histone modification marks at the AR binding site of the miR-221/-222 locus versus that of the PSA locus (Fig 3a). ChIP-qPCR was performed in both LNCaP and C4-2B cells in the presence or absence of androgen. We observed enrichment of H3K9/14 (general) and H3K27 (enhancer-specific) acetylation at both loci (Fig 3b). Acetylation levels were significantly increased after androgen stimulation at the PSA locus, but remained unchanged or slightly decreased at the miR-221/-222 locus. H3K4me2 and H3K4me3 have been the most efficient marker for AR enhancer and promoter [38]. We found H3K4me2 and me3 are highly enriched at both AR binding sites. Interestingly, both H3K4me2 and me3 decreased with androgen stimulation at the PSA and miR-221/-222 loci. These changes are site-specific rather than global likely due to nucleosome repositioning or epitope masking [39]. H3K27me3, an inactive marker for transcription, was also decreased at both AR binding sites. Furthermore, we examined Pol II occupancy at the promoter region and AR binding site. The Pol II level was similar between the PSA and miR-221/-222 genes in the absence of androgen at the promoter (Fig 3c). The association of Pol II at the PSA promoter was significantly increased after androgen stimulation. In contrast, androgen treatment reduced the binding of Pol II to the promoter of miR-221/-222. Similar changes of Pol II occupancy were observed at AR binding sites. These results indicate AR-mediated activation and repression are associated with histone H3 acetylation level, which either facilitates or blocks Pol II recruitment to the TSS.

### AR overexpression could provide a possible explanation for downregulation of miR-221/-222 in CRPC

**Fig 4:**
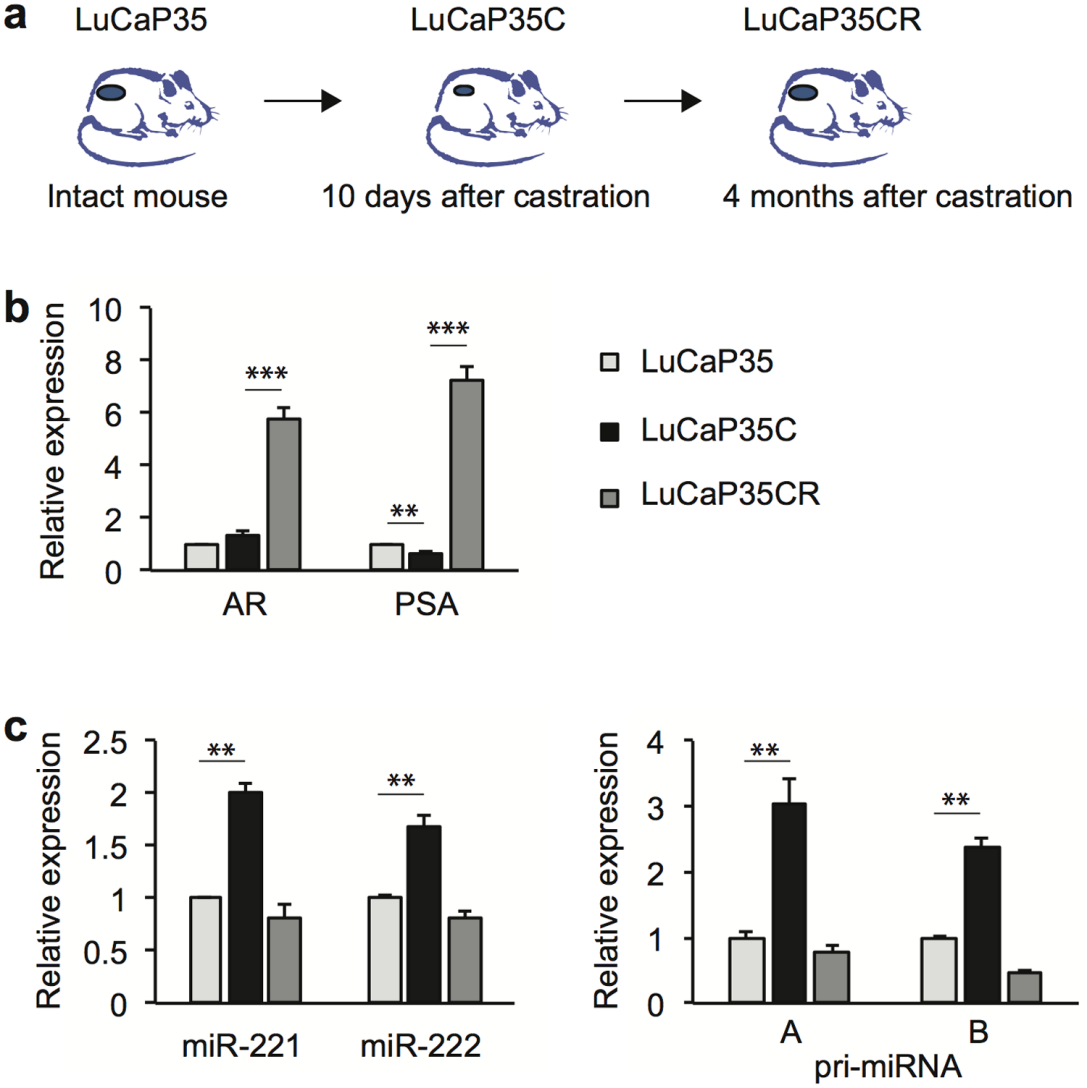
Expression of AR, PSA, miR-221/-222 in xenograft tissues. Total RNA was extracted from fresh frozen tumor tissues and examined for gene expression using RT-qPCR. The p-value was determined using a two-tailed Student’s t-test. Data presented are mean ± SD of three measurements. ** *P* < 0.01; *** *P* < 0.0001.

**Fig 5:**
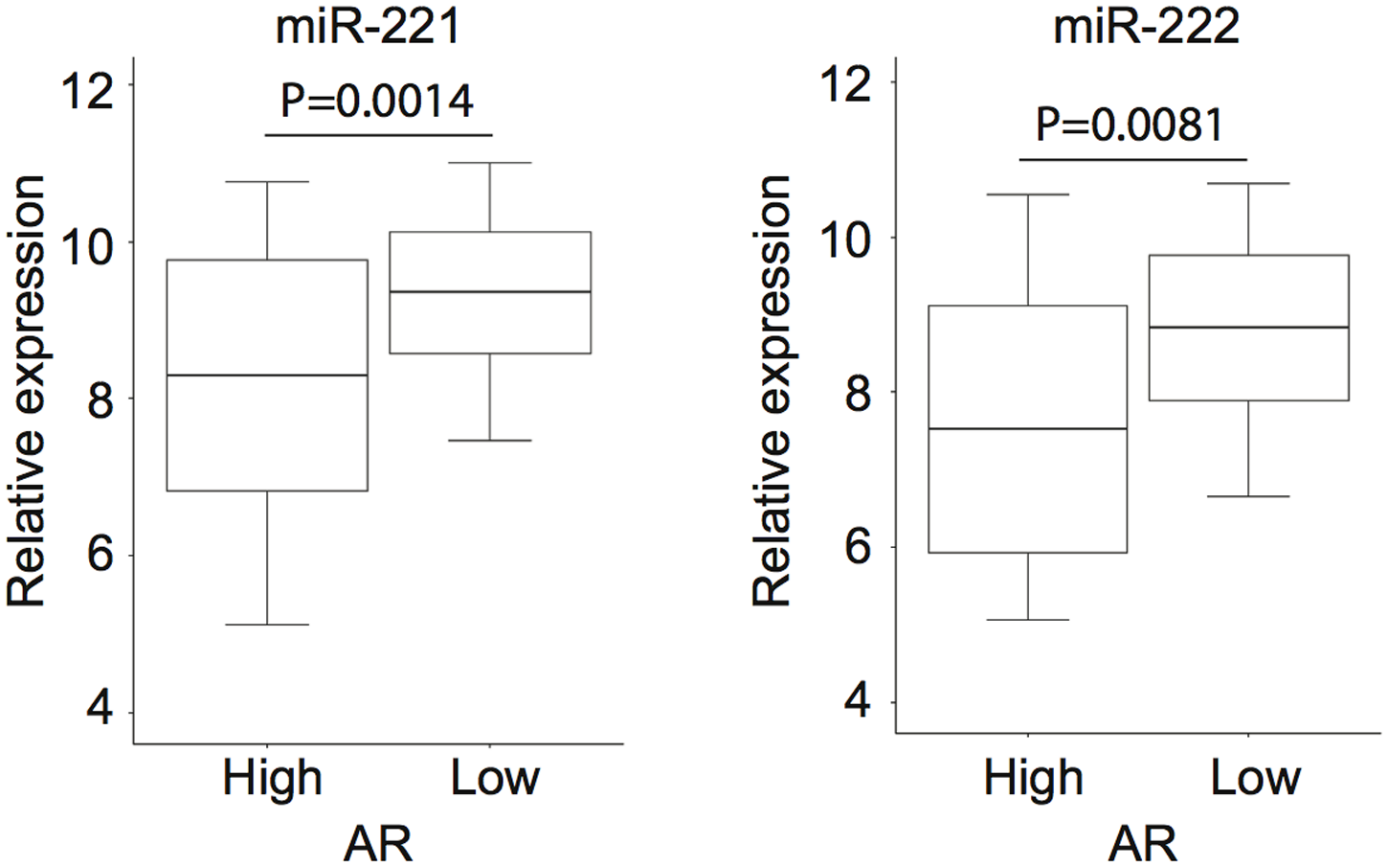
The expression levels of miR-221/-222 and AR are inversely correlated in PCa tumors. Differential expression of miR-221/-222 between high-AR and low-AR groups was analyzed by non-parametric Mann-Whitney test. Boxplot shows the mean ± 95% confidence interval (CI).

While miR-221/-222 are considered as oncogenes in CRPC, it is not clear why their expression level is downregulated in CRPC patients in several studies [11, 16, 20]. Here, we used a xenograft mouse model to mimic clinical CRPC development and progression. LuCaP 35 is an androgen-dependent xenograft, whereas LuCaP 35CR is an androgen-independent variant of LuCaP 35 [30]. LuCaP 35 and LuCaP 35CR xenografts are implanted and maintained in intact and castrated immunodeficient mice respectively (Fig 4a). In order to examine gene expression changes immediate after castration, we obtained LuCaP 35 tumor at day 10 post-castration (referred to as LuCaP 35C). We found that AR target gene PSA expression level was decreased 30% 10 days after castration while the AR level remained unchanged or slightly increased (Fig 4b). Upon emergence of CRPC, both AR and PSA were dramatically overexpressed in the LuCaP 35CR tumor. In contrast, the expression level of mature miR-221/-222 and their primary transcript was significantly upregulated in LuCaP 35C after castration (Fig 4c), which might be necessary for the tumor to survive castration. Subsequently, the expression level was suppressed in line with AR overexpression once LuCaP 35CR tumor was established. Because AR amplification or overexpression is commonly observed in clinical CRPC tumors, this may explain lower expression level of miR-221/-222 in these patients. We next examined publically available miRNA expression profiling datasets [40]. Patients were grouped based on whether they have low or high AR expression in their tumors (Fig 5). We found that the expression level of miR-221/-222 is significantly lower in PCa tissues with high AR expression compared with those with low AR expression.

## Discussion

The molecular mechanisms by which PCa cells progress from androgen-dependent to castration-resistant status have been extensively studied. Much of the previous work has been focused on how AR activity is restored through AR overexpression, amplification, mutations and AR variants, which leads to reactivation of some AR-stimulated genes including PSA. Nevertheless, emerging evidence has suggested that one of the molecular mechanisms for PCa cells to survive ADT is derepression of AR-repressed genes that contribute to androgen synthesis, DNA synthesis, and cellular proliferation [41]. While AR-repressed genes including miRNAs have been identified, these genes are not well studied. Here, we showed that AR negatively regulates the expression of miR-221/-222. MiR-221/-222 are expressed from the intergenic region. Thus, precise annotation of the primary transcript for miR-221/-222 is critical for understanding the mechanisms through which miR-221/-222 expression is regulated. Using genomic analysis, we identified a long pri-miRNA of miR-221/-222 in CRPC C4-2B cells. We further characterized its epigenetic features at the promoter and AR enhancer. Our results are consistent with a recent report in which the authors elegantly mapped cell type-specific TSS and regulatory domains for pri-miRNAs using genomic approaches [42]. Specifically, they defined a distal active TSS of pri-miRNA at the miR-221/-222 locus in LNCaP cells, which perfectly matches the TSS of the pri-miRNA in our C4-2B cells.

Our studies revealed that AR-mediated repression is associated with suppression of histone H3 acetylation and pol II binding at this locus. Repressive effects of AR on miR-221/-222 expression restrains their oncogenic potential. ADT may unlock miR-221/-222, which in turn promote G1/S transition through downregulation of cell cycle genes, such as p27Kip1. Recent studies further suggested HECTD2 and RAB1A as miR-221/-222 targets [33]. Downregulation of HECTD2 and RAB1A promotes androgen-independent PCa cell growth. Our results are consistent with the notion that CRPC cells may acquire a dependency after ADT on AR-repressed genes that are otherwise non-essential for androgen-dependent cells. CRPC cells require high activity of miR-221/-222 to maintain sufficient proliferative ability. This is supported by the evidence that miR-221/-222 are implicated in aggressive PCa [21, 35, 43]. Upregulation of miR-221/-222 has been observed in CRPC cell lines and some patients [18, 32]. Furthermore, our results indicate that miR-221/-222 may play a critical role in promoting PCa cell proliferation in the early stage of CRPC. This function will be attenuated once AR activity is restored or AR overexpression occurs in CRPC. We have observed upregulation of miR-221/-222 in PCa xenograft tumors after castration followed by downregulation of miR-221/-222 in AR-overexpressing CRPC tumors. This indicates that the oncogenic role of miR-221/-222 is likely transient, and reactivated AR-mediated pathways may eventually take over to support the continuous growth of PCa cells.

Several studies showed downregulation of miR-221/-222 in metastatic PCa and CRPC specimens, suggesting a tumor suppressor role for miR-221/-222 [11, 13, 16]. However, miR-221/-222 are not always tumor-suppressive in functional analyses. The effect of miR-221/-222 on PCa cell growth is cell type-specific and context-dependent. Kneitz et al showed that overexpression of miR-221 inhibits AR-negative PC3 and DU145 cell proliferation, but has no inhibitory effect on AR-positive LNCaP cells [13]. Goto et al reported that overexpression of miR-221/-222 has no effect on PC3 and DU145 cell growth, but promotes their migration and invasion *in vitro* [11]. In contrast, Galardi et al showed that overexpression of miR-221/-222 in LNCaP cells strongly induces cell growth while knockdown of miR-221/-222 in PC3 cells reduces their colony formation *in vitro* [9]. Mercatelli used *in vivo* approaches and confirmed that overexpression of miR-221 in LNCaP cells confers a high growth advantage and inhibition of miR-221/-222 in PC3 cells reduces tumor growth in mice [15]. These observed differences indicate a specific function of miR-221/-222 in different phases of PCa development and progression, which can be achieved by involving different targets since one miRNA may regulate multiple genes. In this study, we showed oncogenic role of miR-221/-222 in CRPC C4-2B cells through promoting G1/S phase transition. We further demonstrated that miR-221/-222 are AR-repressed genes. Overexpression of AR may explain downregulation of miR-221/-222 expression in CRPC. Although our results may only reflect a small set of CRPC patients, our finding are consistent with the notion that derepression of miR-221/-222 after ADT is critical for the continuous growth of PCa cells when AR signaling is blocked. MiR-221/-222 are potential therapeutic targets for certain patients during CRPC development.

## Conclusions

MiR-221/-222 are well-established oncogenes in all epithelial cancers except prostate cancer (PCa). In the present study, we show miR-221/-222 are AR-repressed genes and their expression and oncogenic function are associated with AR status in PCa cells. The findings provide an explanation of why miR-221/-222 act as oncogenes in the development of CRPC, but their overexpression is not observed in CRPC tumors. Our findings shed light on the complexity of transcriptional regulation of miR-221/-222 in PCa and suggest context-dependent targeting of oncogenic miR-221/-222.

## Supporting information

S1 Table MicroRNA expression microarray data. (XLSX)
